# Perceived stress and coping strategies used by undergraduate dental students: An observational study

**DOI:** 10.1371/journal.pone.0318152

**Published:** 2025-01-27

**Authors:** Saira Atif, Nilofar Mustafa, Sarah Ghafoor

**Affiliations:** 1 Department of Medical Education, MHPE Scholar, University of Health Sciences, Lahore, Pakistan; 2 Combined Military Hospital Lahore Medical College & Institute of Dentistry, Lahore, Pakistan; 3 National University of Medical Sciences, Rawalpindi, Pakistan; 4 Department of Oral Biology, University of Health Sciences, Lahore, Pakistan; King Abdulaziz University Faculty of Medicine, SAUDI ARABIA

## Abstract

Studies around the world have reported that dental students experience higher stress compared to medical students. Prolonged and high perceived stress can be of a significant concern as it affects the personal, psychological, and professional well-being of the student, affecting quality of life. The aim of the study was to describe the perceived stress and coping strategies that undergraduate students at dental schools of Lahore, Pakistan employ. This observational study was conducted in year 2023 to report perceived stress and coping strategies among 720 undergraduate dental students of Lahore using modified Dental Environment Stress (DES) and brief Coping Orientation to Problems Experienced (COPE) questionnaires. Categorical variables such as sex, year of study (year 1 till 4), and responses to DES stress domains, stress-related items, COPE domains, and COPE-related items were computed into frequencies and percentages. Mean item scores of stress domains, stress-related items, COPE domains, and COPE-related items were presented with standard deviations (SD). Groups wise comparisons were done using Mann Whitney U and Kruskal Wallis H tests. P value < 0.05 was considered statistically significant. The majority of the undergraduate dental students perceived moderate amount of stress. Item “overload feeling due to huge syllabus” had the highest mean stress score (3.49±0.76) and “language barrier” had the lowest mean stress score (1.75±0.92). Female students had higher mean stress scores than the male students in all of the DES domains. Mean stress scores were higher in year 4 students for “workload”, “self-efficacy beliefs”, “faculty and administration”, “clinical training”, and “social stresses” compared to other years. “Religion” was the highest- and “denial” was the lowest-ranked coping strategies. Female students employed “active coping”, “positive reframing”, “religion”, “venting”, “self-blame”, “denial”, and “behavioral disengagement” coping strategies more than males. Moreover, final year dental students used “instrumental support”, “humor”, “self-blame”, “denial”, and “behavioral disengagement” coping strategies more than year 1 and 2 students. Sex and year of study can influence the degree of perceived stress and choice of coping strategies employed to overcome stressful situations. For the emotional, psychological, and professional well-being of undergraduate students, institutions must provide a nurturing and positive learning environment implementing strategies for stress prevention and management.

## Introduction

Stress is a state of mind which is caused by a stressor which can be physical or psychological in nature disturbing the normal physiological balance of the human body [1]. Human body perceives stress as a threat to the emotional health. Humans respond to stress through different emotions which can be anger, fear, anxiety, depression, guilt, or sadness [2]. Hence, coping strategies are developed to deal with the stressors for the emotional well-being to re-gain the equilibrium [3]. It has been suggested that stress itself is not harmful to the physiological and psychological health; it is the inability to effectively cope with the stress that causes emotional and physical problems and, in some cases, may lead to unhealthy habits such as smoking, alcoholism, eating disorders, and addiction [4].

Students have always been a population vulnerable to stressful situations due to their pursuit of professional achievements in a competitive learning environment [5]. The dentistry school curriculum requires students to grasp a variety of theoretical, clinical, and patient-related knowledge and skills, leading to a demanding lifestyle that is detrimental to one’s physical and emotional health [6]. Over 50% students studying medicine and dentistry reported feeling stressed [7]. Evidence suggests that dental students experience considerable amount of stress during their educational years in the dental schools that affects their academic performance, general health, and behavioral responses [8,9]. Additionally, stress can impair dental students’ professional effectiveness by shortening their attention span, impairing their ability to make decisions, and hindering their capacity to form positive relationships with teachers, clinician trainers, or patients [10].

Coping is linked to stress as an adaptation feature as it moderates the impact of stress. It has been suggested that coping techniques and skills are fundamental to prevent stress from getting individuals down and to help them to thrive in challenging times [6]. There are different methods of coping with stress which depend on individual’s personality and preference. Dental students who undergo extreme stress during their educational years have mental, physical, and social repercussions and poor stress coping strategies compared to students who perceive low stress because of using effective coping strategies [11].

Dental students are a vital component of any team of health professionals, and their educational experiences affect their general health, psychosocial well-being, clinical abilities, and academic achievement. High perceived stress have been reported among Pakistani undergraduate medical students [12–15], dental students [8], and postgraduate dental students [16]. The undergraduate dental students’ perception of stress can vary based on demographic factors such as age, sex, marital status, and academic year of study [6,17–19]. Moreover, several factors such as sex, ethnicity, culture, and socioeconomic status can affect the coping strategies used to manage stress. Intensive research has been done to identify stressors which affect the emotional and psychological well-being of students and different coping mechanisms have been identified which are commonly utilized by the students. However, there are limited published studies on perceived stress and coping strategies used by dental undergraduate students in Lahore, Pakistan. Cultural norms, environmental factors, educational experiences, and social demography are different in different cities and even in different dental schools of the same city which demands a more thorough understanding of perceived stress and coping strategies. It is, therefore, necessary to explore perceived stress and any coping strategies employed by the dental students to better understand their educational experience and improve the quality of their educational experience. The objectives of this study were (1) To describe the perceived stress and coping strategies used by the undergraduate dental students using Dental Environment Stress (DES) and brief-Coping Orientation to Problems Experienced (COPE) questionnaires, respectively. (2) To compare stress and coping strategies between male and female students and between year of study of dental students of Lahore, Pakistan.

## Materials and methods

### Study design and participants

This cross-sectional observational study was conducted in five out of ten dental schools of Lahore, Pakistan selected through simple random sampling method from 15^th^ August till 15^th^ December 2023. The minimum sample size was calculated by reference from previous published study [8] considering 95% confidence level, power of study as 90%, 10% non-response rate, anticipated mean DES score of final year (2.61±0.99) and first year (1.66±0.88) dental students. The minimum calculated sample size was 22 students from each year from each dental school. For comparison between male and female dental students, the minimum sample size was calculated by reference from previous published study [8], considering 95% confidence level, power of study as 80%, 10% non-response rate, anticipated mean DES score of female (3.16±0.83) and male (2.72±0.71) dental students. The required sample size was 54 male and 54 female students from each year per college. As the number of male students enrolled at dental colleges in Lahore, Pakistan was less than 25% of the class strength, finite population correction was applied. Hence, the calculated sample size was 12 male and 12 female students (24 students) per year from each dental college. It was decided to include 36 students per year from each school to avoid fall-out and improve reliability. Students were selected through simple random sampling using computer generated random numbers using www.calculatorsoup.com. Students who migrated from other dental schools and repeaters were excluded from the study.

### Ethics statement

The entire study was conducted in accordance with the Declaration of Helsinki, and ethical approval was obtained from Ethical Review Committee (UHS/REG-23/ERC/3136). Approvals were also obtained from the head of the of the five dental schools and the students were approached during school time for explanation of study purpose. Participants were reassured that the confidentiality of their replies would be maintained by the principal investigator and that participation was entirely on a voluntary basis. Queries of the participants were addressed. After that, consent forms were distributed to the students. The participant information sheet and questionnaires did not ask for any personal details of the participants, such as names, class roll numbers, address, or name of dental school, keeping identity of the respondents as anonymous.

After informed written consent, the questionnaires were shared with the participants through Google Forms. In Google form design, all questions were mandatory to answer. If any participant withdrew from the study or was absent on the day of data collection, another participant was selected through random number method to meet the minimum sample size requirement. The average time spent filling the questionnaire was calculated as 15 minutes. The first part of the questionnaire comprised of demographic details of the participants as sex, year of study, marital status, and current living arrangements; whereas the second part consisted of response items related to perceived stress and coping strategies.

### Assessment of perceived stress

For perceived stress, a modified and pre-validated DES scale consisting of 42 stress-related items was used [20–23]. Twelve year 4 dental students from the same college who were randomly chosen to represent the total research population participated in a pilot study. Pre-testing was done to determine whether the study design and questionnaire were adequate and to reveal any practical issues that might arise during the study execution. Students were asked to rate their responses regarding their perceptions to the questionnaire items on a four-point Likert scale ranging from 1: “not stressful at all”, 2: “somewhat stressful”, 3: “quite stressful”, 4: “very stressful” [23]. The scale “not applicable” was added before responses were collected from all study participants, after review of literature and pilot testing, because some of the items related to clinical aspects were not applicable to the students of pre-clinical years [6,20].

### Assessment of coping strategies

The coping strategies that have been proposed most appropriate to combat stress were evaluated using the brief COPE questionnaire, popularly utilized as a behavioral self-regulation model [24]. The responses anticipated from participants were based on their reaction to different stressful circumstances in the dental learning environment that were tabulated on a three-point Likert scale. Response choices ranged from 1: “I have not been doing this at all” to 3: “I have been doing this a lot”. It was expected that the students would make their choices according to the coping tactics that they used most frequently to manage the stressful events experienced by them in dental school. Problem-focused coping comprised “active coping, planning, and use of instrumental support”. Emotion-focused coping consisted of “use of emotional support, positive reframing, humor, acceptance, religion, venting, and self-blame”. Avoidant coping consisted of the following COPE domains: “distraction, denial, and behavioral disengagement”.

### Data analyses

Data were analyzed with SPSS version 27.0. Sex, year of study, marital status, and living arrangement were computed as frequencies and percentage. Stress-related items and cope-related items were computed into frequencies and percentages and mean items scores were presented with standard deviation (SD) as appropriate. Stress-items having mean scores of < 2 were considered as ‘mildly stressful’, between 2 & < 3 as ‘moderately stressful’ and those either 3 or > 3 as ‘highly stressful’ [8]. These were presented as frequency and percentages. The means of all the stress domains and coping strategies were also presented as means with SDs as the data were normally distributed as per Shapiro-Wilk’s test. As the data were in Likert response format, Mann Whitney U tests were used to compare stress domains and stress items of DES with sex, and brief-COPE domains and items with sex. Kruskal Wallis H tests were used to compare year of study with stress domains and stress items of DES, and with brief-COPE domains and items. In all these tests, p value < 0.05 was considered statistically significant.

### Results

A total of 720 completely filled questionnaires were received, 180 from each year of study. The overall reliability of DES questionnaire with 42 items was assessed by inter-item correlation though Cronbach’s alpha, the value came out to be 0.92 (excellent internal consistency). The reliability of brief COPE questionnaire with 26 items assessed by inter-item correlation using Cronbach’s alpha was 0.85 (good internal consistency). The mean age of study participants was 20.84±1.56 years. There were 534 (74.2%) females and 186 (25.6%) males who participated in this study. All the participants were single and never married. The majority of the participants 389 (54%) were living with their parents at home, 318 (44.2%) were living in dormitories, and the remaining 13 (1.8%) were living with their relatives at home.

### Responses of participants to DES questionnaire

It was noted that out of 42 items, 36 (85.7%) items were found to be moderately stressful as shown in Table 1. Moreover, in the domain of “workload”, item “overloaded feeling due to huge syllabus” was highly stressful in majority 459 (63.8%) of the students. In “self-efficacy” domain “fear of failing a course or the year” was reported as being highly stressful in the majority of the students 482 (66.9%), as given in Table 1.

**Table 1:** DES questionnaire with mean scores and ranks of the stress-related items.

Stress-related item		Level of stress n (%)					Mean item score ±SD	Rank
		Not stressful at all	Somewhat stressful	Quite stressful	Very stressful	Not applicable		
**Workload domain**							2.86±0.63	
1	Amount of assigned class work	94 (13.1)	287 (39.9)	226 (31.4)	113 (15.7)	-	2.50±0.91	7
2	Lack of time to do assigned work	53 (7.4)	273 (37.9)	232 (32.2)	162 (22.5)	-	2.70±0.89	5
3	Difficulty of understanding course material/lecture	69 (9.6)	292 (40.6)	253 (35.1)	106 (14.7)	-	2.55±0.86	6
4	Late ending day	60 (8.3)	193 (26.8)	230 (31.9)	237 (32.9)	-	2.89±0.96	3
5	Lack of time for relaxation†	41 (5.7)	153 (21.3)	251 (34.9)	275 (38.2)	-	3.06±0.91	2
6	Overloaded feeling due to huge syllabus†	14 (1.9)	78 (10.8)	169 (23.5)	459 (63.8)	-	3.49±0.76	1
7	Lack of time between seminars and laboratories or clinics	89 (12.4)	186 (25.8)	180 (25)	265 (36.8)	-	2.86±1.05	4
**Self-efficacy beliefs domain**							2.75±0.74	
8	Fear of failing a course or the year†	40 (5.6)	81 (11.3)	117 (16.3)	482 (66.9)	-	3.45±0.89	1
9	Fear of being unable to catch up if behind†	34 (4.7)	115 (16)	190 (26.4)	381 (52.9)	-	3.28±0.89	2
10	Lack of confidence to be a successful dental student	103 (14.3)	181 (25.1)	157 (21.8)	279 (38.8)	-	2.85±1.09	5
11	Fear of not being able to join a post-graduate dental education program	105 (14.6)	211 (29.3)	190 (26.4)	214 (29.7)	-	2.71±1.05	6
12	Insecurity concerning professional future	72 (10)	178 (24.7)	240 (33.3)	228 (31.7)	2 (0.3)	2.87±0.98	4
13	Insecurity concerning lack of employment positions	76 (10.6)	183 (25.4)	174 (24.2)	287 (39.9)	-	2.93±1.04	3
14	Lack of confidence in own decision making	142 (19.7)	242 (33.6)	173 (24)	163 (22.6)	-	2.50±1.05	7
15	Language barrier	358 (49.7)	236 (32.8)	71 (9.9)	55 (7.6)	-	1.75±0.92	9
16	Lack of confidence to be a successful dentist	149 (20.7)	238 (33.1)	195 (27.1)	138 (19.2)	-	2.45±1.02	8
**Faculty and administration domain**							2.31±0.65	
17	Inconsistency of feedback on work between different instructors	119 (16.5)	314 (43.6)	184 (25.6)	103 (14.3)	**-**	2.38±0.92	4
18	Receiving criticism about work	168 (23.3)	248 (34.4)	225 (31.3)	79 (11)	-	2.30±0.95	6
19	Being treated as immature & irresponsible by faculty	132 (18.3)	169 (23.5)	224 (31.1)	195 (27.1)	-	2.67±1.06	1
20	Availability of qualified laboratory technicians	236 (32.8)	122 (16.9)	73 (10.1)	103 (14.3)	186 (25.8)	2.08±1.16	8
21	Lack of input into the decision-making process of the school	138 (19.2)	248 (34.4)	185 (25.7)	149 (20.7)	-	2.48±1.02	2
22	Getting study material	136 (18.9)	290 (40.3)	159 (22.1)	135 (18.8)	-	2.41±1.00	3
23	Shortage of allocated laboratory time	233 (32.4)	190 (26.4)	173 (24)	124 (17.2)	-	2.26±1.09	7
24	Inadequate number of instructors in relation to student	242 (33.6)	271 (37.6)	129 (17.9)	78 (10.8)	-	2.06±0.97	9
25	Shortage of allocated clinical time	123 (17.1)	164 (22.8)	102 (14.2)	98 (13.6)	233 (32.4)	2.36±1.07	5
26	Amount of cheating in dental school	260 (36.1)	233 (32.4)	138 (19.2)	89 (12.4)	-	2.08±1.02	8
**Patient treatment/interaction domain**							2.53±0.86	
27	Patients being late or not showing for their appointments	60 (8.3)	136 (18.9)	109 (15.1)	101 (14)	314 (43.6)	2.62±1.02	2
28	Lack of cooperation by patients in their home care	122 (16.9)	143 (19.9)	113 (15.7)	80 (11.1)	262 (36.4)	2.33±1.05	4
29	Fear of dealing with patients who do not disclose the existence of a contagious / co-existing disease	41 (5.7)	125 (17.4)	149 (20.7)	128 (17.8)	277 (38.5)	2.82±0.96	1
30	Working on patients with dirty mouths	89 (12.4)	152 (21.1)	95 (13.2)	119 (16.5)	265 (36.8)	2.54±1.08	3
**Clinical training domain**							2.55±0.87	
31	Responsibility of getting suitable patients	97 (13.5)	139 (19.3)	112 (15.6)	106 (14.7)	266 (36.9)	2.50±1.07	4
32	Difficulty in learning precision manual skills required in pre-clinical work	82 (11.4)	176 (24.4)	134 (18.6)	110 (15.3)	218 (30.3)	2.54±1.01	3
33	Transition from pre-clinic to clinical work	64 (8.9)	122 (16.9)	132 (18.3)	111 (15.4)	291 (40.4)	2.68±1.02	1
34	Difficulty in learning clinical procedures	85 (11.8)	149 (20.7)	141 (19.6)	113 (15.7)	232 (32.2)	2.58±1.03	2
**Performance pressure domain**							2.77±0.71	
35	Examination and quizzes	44 (6.1)	171 (23.8)	257 (35.7)	248 (34.4)	-	2.98±0.91	1
36	Competition for grades	62 (8.6)	176 (24.4)	238 (33.1)	244 (33.9)	-	2.92±0.96	2
37	Clinical requirements	135 (18.8)	267 (37.1)	210 (29.2)	108 (15)	-	2.40±0.96	3
**Social stresses domain**							2.32±0.79	
38	Lack of home environment in living quarters	214 (29.7)	160 (22.2)	148 (20.6)	198 (27.5)	-	2.46±1.18	2
39	Financial responsibilities	90 (12.5)	176 (24.4)	233 (32.4)	221 (30.7)	-	2.81±1.01	1
40	Forced postponement of engagement or marriage	388 (53.9)	154 (21.4)	65 (09)	113 (15.7)	-	1.87±1.11	5
41	Martial adjustment problems	147 (20.4)	49 (6.8)	42 (5.8)	58 (8.1)	424 (58.9)	2.04±1.19	4
42	Necessity to postpone having children	121 (16.8)	68 (9.4)	39 (5.4)	56 (7.8)	436 (60.6)	2.11±1.16	3

Within each stress domain, the stress-related items are ranked from those having the highest mean score to the lowest one having the least mean score. Items with

† are ones having mean scores ≥ 3 ‘highly stressful’.

It was found that female students had higher mean scores than the male students in all seven stress domains. In 34 out of 42 DES items, there were statistically significant associations with sex indicating that majority of female students perceived more stress compared to male students (Table 2).

**Table 2:** Comparison of sex and DES domain and item scores.

Stress domain	Sex	Mean domain score±SD	p value	Stress item	Sex	Mean item score±SD	p value
**Workload**	F	2.94±0.59	**<0.001** *	Amount of assigned class work	F	2.61±0.91	**<0.001** *
	M	2.64±0.68			M	2.18±0.83	
				Lack of time to do assigned work	F	2.81±0.90	**<0.001** *
					M	2.38±0.81	
				Difficulty of understanding course material/lecture	F	2.62±0.84	**<0.001** *
					M	2.34±0.88	
				Late ending day	F	2.96±0.89	**0.016** *
					M	2.71±1.11	
				Lack of time for relaxation	F	3.12±0.87	**0.005** *
					M	2.88±0.98	
				Overloaded feeling due to huge syllabus	F	3.56±0.69	**<0.001** *
					M	3.28±0.92	
				Lack of time between seminars and laboratories or clinics	F	2.91±1.02	0.057
					M	2.72±1.13	
**Self-efficacy beliefs**	F	2.87±0.72	**<0.001** *	Fear of failing a course or the year	F	3.54±0.79	**<0.001** *
	M	2.43±0.70			M	3.17±1.12	
				Fear of being unable to catch up if behind	F	3.37±0.82	**<0.001** *
					M	2.99±1.04	
				Lack of confidence to be a successful dental student	F	3.01±1.05	**<0.001** *
					M	2.38±1.08	
				Fear of not being able to join a post-graduate dental education program	F	2.85±1.03	**<0.001** *
					M	2.32±0.98	
				Insecurity concerning professional future	F	2.92±0.97	0.056
					M	2.74±0.99	
				Insecurity concerning lack of employment positions	F	3.0±1.01	**0.003** *
					M	2.73±1.08	
				Lack of confidence in own decision making	F	2.65±1.06	**<0.001** *
					M	2.04±0.89	
				Language barrier	F	1.85±0.96	**<0.001** *
					M	1.48±0.72	
				Lack of confidence to be a successful dentist	F	2.6±1.04	**<0.001** *
					M	2.02±0.86	
**Faculty and administration**	F	2.37±0.63	**<0.001** *	Inconsistency of feedback on work between different instructors	F	2.45±0.92	**<0.001** *
	M	2.12±0.68			M	2.17±0.89	
				Receiving criticism about work	F	2.41±0.94	**<0.001** *
					M	1.97±0.89	
				Being treated as immature & irresponsible by faculty	F	2.79±1.03	**<0.001** *
					M	2.33±1.09	
				Availability of qualified laboratory technicians	F	2.07±1.17	0.512
					M	2.13±1.15	
				Lack of input into the decision-making process of the school	F	2.56±1.05	**0.001** *
					M	2.25±0.93	
				Getting study material	F	2.47±1.01	**0.007** *
					M	2.24±0.95	
				Shortage of allocated laboratory time	F	2.34±1.08	**0.001** *
					M	2.03±1.08	
				Inadequate number of instructors in relation to student	F	2.13±1.01	**0.002** *
					M	1.84±0.83	
				Shortage of allocated clinical time	F	2.39±1.09	0.300
					M	2.26±0.98	
				Amount of cheating in dental school	F	2.07±1.04	0.583
					M	2.10±0.98	
**Patient treatment/interaction**	F	2.63±0.83	**<0.001** *	Patients being late or not showing for their appointments	F	2.70±0.98	**0.011** *
	M	2.22±0.89			M	2.38±1.09	
				Lack of cooperation by patients in their home care	F	2.40±1.06	**0.010** *
					M	2.09±0.99	
				Fear of dealing with patients who do not disclose the existence of a contagious / co-existing disease	F	2.90±0.89	**0.008** *
					M	2.55±1.11	
				Working on patients with dirty mouths	F	2.67±1.04	**<0.001** *
					M	2.04±1.09	
**Clinical training**	F	2.65±0.83	**<0.001** *	Responsibility of getting suitable patients	F	2.56±1.06	**0.015** *
	M	2.21±0.94			M	2.27±1.09	
				Difficulty in learning precision manual skills required in pre-clinical work	F	2.62±0.98	**0.001** *
					M	2.27±1.07	
				Transition from pre-clinic to clinical work	F	2.81±0.96	**<0.001** *
					M	2.17±1.09	
				Difficulty in learning clinical procedures	F	2.67±0.99	**<0.001** *
					M	2.24±1.08	
**Performance pressure**	F	2.89±0.64	**<0.001** *	Examination and quizzes	F	3.08±0.84	**<0.001** *
	M	2.40±0.77			M	2.71±1.03	
				Competition for grades	F	3.09±0.91	**<0.001** *
					M	2.43±0.94	
				Clinical requirements	F	2.52±0.93	**<0.001** *
					M	2.07±0.96	
**Social stresses**	F	2.38±0.80	**<0.001** *	Lack of home environment in living quarters	F	2.60±1.16	**<0.001** *
	M	2.15±0.77			M	2.06±1.14	
				Financial responsibilities	F	2.90±1.00	**<0.001** *
					M	2.56±0.99	
				Forced postponement of engagement or marriage	F	1.86±1.17	0.103
					M	1.88±0.95	
				Martial adjustment problems	F	2.07±1.19	0.207
					M	1.93±1.22	
				Necessity to postpone having children	F	2.08±1.13	0.723
					M	1.18±1.24	

F: Female; M: Male; Mann Whitney U test applied

*p < 0.05 has been taken as significant.

Comparisons were done between year of study and DES domains as given in Table 3. Among the stress domains, significant associations were seen with the year of study in six out of seven domains. Mean stress domain scores were lower for “workload” in year 3 students compared to other years. Mean stress scores were higher in year 4 students for “self-efficacy”, “faculty and administration”, “clinical training”, and “social stresses” domains compared to other years. In year 1 students, domains with higher mean stress scores were “patient treatment/interaction” and “clinical training” compared to other years. Significant associations were seen in the majority of the items with year of study as given in Table 3. Year 1 students had higher mean item stress scores compared to other years of study for the following items: “amount of assigned class work”, “lack of time to do assigned work”, “overloaded feeling due to huge syllabus”, “working on patients with dirty mouths”, and “examination and quizzes”.

**Table 3:** Comparison of year of study and DES stress-items.

DES domain	Year of study	Mean domain score±SD	p value	Stress item	Year of study	Mean item score±SD	p value
**Workload**	1	2.93±0.52	**<0.001** *	Amount of assigned class work	1	2.73±0.89	**<0.001** *
	2	2.93±0.69			2	2.52±0.92	
	3	2.66±0.66			3	2.22±0.92	
	4	2.94±0.58			4	2.52±0.83	
				Lack of time to do assigned work	1	2.89±0.80	**<0.001** *
					2	2.75±0.83	
					3	2.36±0.92	
					4	2.80±0.95	
				Difficulty of understanding course material/lecture	1	2.63±0.87	0.056
					2	2.47±0.87	
					3	2.46±0.91	
					4	2.64±0.75	
				Late ending day	1	2.86±0.96	**0.003** *
					2	2.97±0.89	
					3	2.68±1.05	
					4	3.07±0.89	
				Lack of time for relaxation	1	3.09±0.89	**<0.001** *
					2	3.29±0.79	
					3	2.83±1.01	
					4	3.01±0.86	
				Overloaded feeling due to huge syllabus	1	3.61±0.64	**0.005** *
					2	3.51±0.86	
					3	3.33±0.84	
					4	3.52±0.67	
				Lack of time between seminars and laboratories or clinics	1	2.69±1.07	**0.003** *
					2	2.97±1.07	
					3	2.76±1.02	
					4	3.03±1.00	
**Self-efficacy beliefs**	1	2.55±0.64	**<0.001** *	Fear of failing a course or the year	1	3.48±0.87	0.202
	2	2.59±0.86			2	3.27±1.05	
	3	2.86±0.68			3	3.52±0.86	
	4	3.02±0.67			4	3.52±0.79	
				Fear of being unable to catch up if behind	1	3.24±0.79	0.155
					2	3.15±1.08	
					3	3.41±0.79	
					4	3.30±0.88	
				Lack of confidence to be a successful dental student	1	2.47±1.11	**<0.001** *
					2	2.66±1.15	
					3	2.96±1.03	
					4	3.32±0.87	
				Fear of not being able to join a post-graduate dental education program	1	2.43±1.09	**<0.001** *
					2	2.57±1.08	
					3	2.79±0.97	
					4	3.06±0.92	
				Insecurity concerning professional future	1	2.57±0.94	**<0.001** *
					2	2.72±1.02	
					3	3.07±0.98	
					4	3.12±0.85	
				Insecurity concerning lack of employment positions	1	2.73±1.08	**<0.001** *
					2	2.53±1.04	
					3	3.28±0.91	
					4	3.18±0.92	
				Lack of confidence in own decision making	1	2.30±1.02	**<0.001** *
					2	2.26±1.04	
					3	2.57±1.09	
					4	2.85±0.93	
				Language barrier	1	1.47±0.75	**<0.001** *
					2	1.79±0.96	
					3	1.77±0.87	
					4	1.98±1.01	
				Lack of confidence to be a successful dentist	1	2.23±1.01	**<0.001** *
					2	2.32±1.07	
					3	2.43±1.06	
					4	2.82±0.85	
**Faculty and administration**	1	2.41±0.55	**<0.001** *	Inconsistency of feedback on work between different instructors	1	2.20±0.80	**<0.001** *
	2	2.49±0.68			2	2.36±0.96	
	3	2.36±0.72			3	2.35±0.98	
	4	2.61±0.58			4	2.60±0.90	
				Receiving criticism about work	1	2.32±0.96	**<0.001** *
					2	1.84±0.81	
					3	2.37±0.96	
					4	2.67±0.87	
				Being treated as immature & irresponsible by faculty	1	2.56±1.05	**0.004** *
					2	2.49±1.13	
					3	2.79±1.09	
					4	2.83±0.94	
				Availability of qualified laboratory technicians	1	1.44±0.73	**<0.001** *
					2	1.59±0.98	
					3	2.07±1.09	
					4	2.68±1.19	
				Lack of input into the decision-making process of the school	1	2.17±0.92	**<0.001** *
					2	2.35±1.08	
					3	2.66±1.01	
					4	2.74±0.98	
				Getting study material	1	2.46±1.05	**<0.001** *
					2	2.17±0.96	
					3	2.37±1.02	
					4	2.65±0.91	
				Shortage of allocated laboratory time	1	1.76±0.98	**<0.001** *
					2	2.14±1.10	
					3	2.34±0.96	
					4	2.80±1.05	
				Inadequate number of instructors in relation to student	1	1.79±0.91	**<0.001** *
					2	2.19±1.05	
					3	1.96±0.93	
					4	2.30±2.06	
				Shortage of allocated clinical time	1	2.13±0.98	**0.027** *
					2	2.16±0.96	
					3	2.34±1.06	
					4	2.53±1.12	
				Amount of cheating in dental school	1	1.83±1.10	**<0.001** *
					2	2.09±0.92	
					3	2.07±0.99	
					4	2.32±1.01	
**Patient treatment / interaction**	1	2.93±0.96	**<0.001** *	Patients being late or not showing for their appointments	1	2.41±1.08	**<0.001** *
	2	2.17±0.89			2	2.55±0.98	
	3	2.27±0.76			3	2.28±0.97	
	4	2.86±0.77			4	2.97±0.94	
				Lack of cooperation by patients in their home care	1	2.53±1.08	**<0.001** *
					2	1.75±0.84	
					3	2.12±1.01	
					4	2.69±1.01	
				Fear of dealing with patients who do not disclose the existence of a contagious / co-existing disease	1	2.92±1.22	**0.002** *
					2	2.69±0.95	
					3	2.64±0.94	
					4	3.02±0.87	
				Working on patients with dirty mouths	1	3.30±0.99	**<0.001** *
					2	2.77±1.05	
					3	2.11±1.04	
					4	2.72±0.97	
**Clinical training**	1	2.56±0.95	**<0.001** *	Responsibility of getting suitable patients	1	2.48±1.19	**<0.001** *
	2	2.28±0.81			2	2.44±1.18	
	3	2.31±0.81			3	2.11±0.99	
	4	2.93±0.82			4	2.91±0.94	
				Difficulty in learning precision manual skills required in pre-clinical work	1	2.53±1.03	**<0.001** *
					2	2.07±0.94	
					3	2.38±1.00	
					4	2.97±0.88	
				Transition from pre-clinic to clinical work	1	2.43±1.12	**<0.001** *
					2	2.90±0.96	
					3	2.41±0.98	
					4	2.91±0.99	
				Difficulty in learning clinical procedures	1	2.61±1.13	**<0.001** *
					2	2.29±0.87	
					3	2.36±1.00	
					4	2.92±1.01	
**Performance pressure**	1	2.81±0.70	0.228	Examination and quizzes	1	3.37±0.81	**<0.001** *
	2	2.75±0.63			2	2.79±0.88	
	3	2.69±0.77			3	2.92±0.93	
	4	2.82±0.72			4	2.87±0.91	
				Competition for grades	1	3.02±1.02	**0.004** *
					2	3.07±0.89	
					3	2.83±0.93	
					4	2.77±0.96	
				Clinical requirements	1	2.04±0.95	**<0.001** *
					2	2.40±0.91	
					3	2.34±0.93	
					4	2.83±0.88	
**Social stresses**	1	2.24±0.75	**0.011** *	Lack of home environment in living quarters	1	2.60±1.24	0.173
	2	2.27±0.78			2	2.46±1.26	
	3	2.26±0.80			3	2.32±1.14	
	4	2.50±0.84			4	2.45±1.07	
				Financial responsibilities	1	2.59±1.01	**<0.001** *
					2	2.86±1.00	
					3	2.76±1.01	
					4	3.03±0.97	
				Forced postponement of engagement or marriage	1	1.67±1.08	**<0.001** *
					2	1.79±1.01	
					3	1.82±1.08	
					4	2.18±1.22	
				Martial adjustment problems	1	1.88±1.23	**0.003** *
					2	1.64±1.11	
					3	2.02±1.16	
					4	2.30±1.19	
				Necessity to postpone having children	1	2.00±1.18	0.216
					2	1.87±1.09	
					3	2.07±1.15	
					4	2.29±1.18	

Kruskal Wallis test applied

*p < 0.05 has been taken as significant.

### Responses of participants to COPE questionnaire

It was noted that out of 26 coping strategy items, 13 items had a score of < 2, while the remaining 13 items had a score range of 2–2.99. None of the coping strategy items had a score of ≥ 3. The COPE-strategy items with highest scores were related to “religion”: “I’ve been trying to find comfort in my religion or spiritual beliefs” and “I’ve been praying and meditating”. The lowest scored items were related to “denial”: “I’ve been saying to myself this isn’t real” and “I’ve been refusing to believe that it has happened” (Table 4). Overall, the COPE-strategy items belonging to coping strategies “denial” and “behavioral disengagement” were the lowest scored items (rank 19–21) as compared to the rest.

**Table 4:** Mean scores and over-all ranks of COPE-strategy items.

Sr. No	Coping strategy	COPE strategy item	Number of responses n (%)			Mean item score ±SD	Rank
			I have not been doing this at all	I have been doing it sometimes	I have been doing this a lot		
**Problem-focused coping**							
1	**Active coping**	I’ve been concentrating my efforts on doing something about the situation I’m in	69 (9.6)	434 (60.3)	217 (30.1)	2.21±0.59	8
		I’ve been taking action to try to make the situation better	40 (5.6)	401 (55.7)	279 (38.8)	2.33±0.58	3
2	**Planning**	I’ve been trying to come up with a strategy about what to do	59 (8.2)	368 (51.1)	293 (40.7)	2.33±0.62	3
		I’ve been thinking hard about what steps to take	62 (8.6)	327 (45.4)	331 (46)	2.37±0.64	2
3	**Use of instrumental support**	I’ve been getting help and advice from other people	168 (23.3)	382 (53.1)	170 (23.6)	2.0±0.69	10
		I’ve been trying to get advice or help from other people about what to do	185 (25.7)	421 (58.5)	114 (15.8)	1.9±0.64	15
**Emotion-focused coping**							
4	**Use of emotional support**	I’ve been getting emotional support from others	221 (30.7)	341 (47.4)	158 (21.9)	1.91±0.72	14
		I’ve been getting comfort and understanding from someone	205 (28.5)	363 (50.4)	152 (21.1)	1.93±0.70	13
5	**Positive reframing**	I’ve been trying to see it in a different light, to make it seem more positive	127 (17.6)	323 (44.9)	268 (37.2)	2.20±0.72	9
		I’ve been looking for something good in what is happening	89 (12.4)	336 (46.7)	295 (41)	2.29±0.67	4
6	**Acceptance**	I’ve been accepting the reality of the fact that it has happened	95 (13.2)	333 (46.3)	292 (40.6)	2.27±0.68	6
		I’ve been learning to live with it	78 (10.8)	364 (50.6)	278 (38.6)	2.28±0.65	5
7	**Humor**	I’ve been making jokes about it	254 (35.3)	280 (38.9)	186 (25.8)	1.91±0.78	14
		I’ve been making fun of the situation	298 (41.4)	263 (36.5)	159 (22.1)	1.81±0.77	18
8	**Religion**	I’ve been trying to find comfort in my religion or spiritual beliefs	49 (6.8)	253 (35.1)	418 (58.1)	2.51±0.62	1
		I’ve been praying or meditating	33 (4.6)	288 (40)	399 (55.4)	2.51±0.59	1
9	**Venting**	I’ve been saying things to let my unpleasant feelings escape	224 (31.1)	364 (50.6)	132 (18.3)	1.87±0.69	16
		I’ve been expressing my negative feelings	235 (32.6)	354 (49.2)	131 (18.2)	1.86±0.70	17
10	**Self-blame**	I’ve been criticizing myself	208 (28.9)	315 (43.8)	197 (27.4)	1.98±0.75	11
		I’ve been blaming myself for things that happened	222 (30.8)	306 (42.5)	192 (26.7)	1.96±0.76	12
**Avoidant coping**							
11	**Self-distraction**	I’ve been turning to work or other activities to take my mind off things	104 (14.4)	363 (50.4)	253 (35.1)	2.21±0.67	8
		I’ve been doing something to think about it less, such as going to movies, watching TV, reading, daydreaming, sleeping, or shopping	97 (13.5)	367 (51)	256 (35.6)	2.22±0.67	7
12	**Denial**	I’ve been saying to myself “this isn’t real”	379 (52.6)	247 (34.3)	94 (13.1)	1.60±0.71	21
		I’ve been refusing to believe that it has happened	383 (53.2)	242 (33.6)	95 (13.2)	1.60±0.71	21
13	**Behavioral disengagement**	I’ve been giving up trying to deal with it	292 (40.6)	326 (45.3)	102 (14.2)	1.74±0.69	20
		I’ve been giving up the attempt to cope	285 (39.6)	327 (45.4)	108 (15)	1.75±0.70	19

COPE strategy items are ranked from those having the highest mean score to the lowest one having the least mean score.

The means and SD for all coping strategies were calculated. As all the coping strategies had an equal number of COPE-strategy items within them, therefore they were also ranked. It was found that the coping strategy “religion” had the highest mean domain score and “denial” had the lowest mean domain score (Table 5). Means and SDs of overall broad COPE categories of problem-focused, emotion-focused, and avoidant coping were also calculated which were 2.19±0.41, 2.09±0.31, and 1.85±0.43, respectively.

**Table 5:** Means with SDs and ranks of coping strategies.

Coping strategy	Mean domain score±SD	Domain rank
Active coping	2.27±0.51	3
Planning	2.35±0.55	2
Use of instrumental support	1.95±0.61	6
Use of emotional support	1.92±0.64	7
Positive reframing	2.24±0.62	4
Acceptance	2.27±0.59	3
Humor	1.86±0.72	7
Religion	2.51±0.54	1
Venting	1.86±0.56	7
Self-blame	1.97±0.70	5
Self-distraction	2.21±0.57	4
Denial	1.60±0.66	9
Behavioral disengagement	1.75±0.63	8

Coping strategies have been ranked on basis of highest mean score to the lowest mean score.

There were significant associations between sex and the following coping strategies: “active coping”, “positive reframing”, “religion”, “venting”, “self-blame”, “denial”, and “behavioral disengagement” (Table 6). Statistically significant relationships were found between sex and 13 of the COPE items. In all these items, it was found that majority of female students were practicing these COPE-strategy items more compared to the male students (Table 6).

**Table 6:** Comparison of sex with COPE domains and coping strategies items.

COPE domain	Sex	Mean item score±SD	p value	Coping Strategy	Sex	Mean item score±SD	p value
**Active coping**	F	2.30±0.51	**0.002** *	I’ve been concentrating my efforts on doing something about the situation I’m in	F	2.25±0.61	**0.001** *
	M	2.17±0.49			M	2.09±0.53	
				I’ve been taking action to try to make the situation better	F	2.36±0.57	**0.043** *
					M	2.25±0.60	
**Planning**	F	2.37±0.54	0.131	I’ve been trying to come up with a strategy about what to do	F	2.36±0.62	**0.005** *
	M	2.29±0.57			M	2.22±0.61	
				I’ve been thinking hard about what steps to take	F	2.38±0.63	0.849
					M	2.36±0.66	
**Use of instrumental support**	F	1.97±0.62	0.288	I’ve been getting help and advice from other people	F	2.03±0.70	0.054
	M	1.89±0.58			M	1.92±0.63	
				I’ve been trying to get advice or help from other people about what to do	F	1.91±0.66	0.606
					M	1.88±0.58	
**Use of emotional support**	F	1.91±0.66	0.575	I’ve been getting emotional support from others	F	1.91±0.74	0.908
	M	1.94±0.57			M	1.91±0.68	
				I’ve been getting comfort and understanding from someone	F	1.92±0.72	0.433
					M	1.96±0.64	
**Positive reframing**	F	2.27±0.63	**0.007** *	I’ve been trying to see it in a different light, to make it seem more positive	F	2.23±0.74	0.075
	M	2.15±0.59			M	2.13±0.67	
				I’ve been looking for something good in what is happening	F	2.33±0.67	**0.004** *
					M	2.17±0.65	
**Acceptance**	F	2.29±0.60	0.230	I’ve been accepting the reality of the fact that it has happened	F	2.27±0.69	0.995
	M	2.23±0.57			M	2.28±0.63	
				I’ve been learning to live with it	F	2.32±0.64	**0.008** *
					M	2.17±0.67	
**Humor**	F	1.86±0.74	0.941	I’ve been making jokes about it	F	1.89±0.79	0.393
	M	1.84±0.66			M	1.94±0.71	
				I’ve been making fun of the situation	F	1.83±0.79	0.276
					M	1.74±0.70	
**Religion**	F	2.57±0.53	**<0.001** *	I’ve been trying to find comfort in my religion or spiritual beliefs	F	2.55±0.62	**<0.001** *
	M	2.34±0.54			M	2.39±0.62	
				I’ve been praying or meditating	F	2.58±0.56	**<0.001** *
					M	2.29±0.59	
**Venting**	F	1.93±0.54	**<0.001** *	I’ve been saying things to let my unpleasant feelings escape	F	1.93±0.66	**<0.001** *
	M	1.67±0.59			M	1.70±0.75	
				I’ve been expressing my negative feelings	F	1.93±0.71	**<0.001** *
					M	1.64±0.63	
**Self-blame**	F	2.00±0.73	**0.034** *	I’ve been criticizing myself	F	2.05±0.79	**<0.001** *
	M	1.88±0.61			M	1.81±0.65	
				I’ve been blaming myself for things that happened	F	1.96±0.79	0.870
					M	1.95±0.66	
**Self-distraction**	F	2.23±0.58	0.340	I’ve been turning to work or other activities to take my mind off things	F	2.20±0.67	0.483
	M	2.17±0.56			M	2.24±0.67	
				I’ve been doing something to think about it less, such as going to movies, watching TV, reading, daydreaming, sleeping, or shopping	F	2.26±0.67	**0.004** *
					M	2.11±0.63	
**Denial**	F	1.67±0.68	**<0.001** *	I’ve been saying to myself ‘this isn’t real’	F	1.67±0.73	**<0.001** *
	M	1.42±0.54			M	1.41±0.59	
				I’ve been refusing to believe that it has happened	F	1.66±0.74	**<0.001** *
					M	1.42±0.58	
**Behavioral disengagement**	F	1.78±0.63	**0.009** *	I’ve been giving up trying to deal with it	F	1.76±0.70	0.080
	M	1.66±0.63			M	1.66±0.66	
				I’ve been giving up the attempt to cope	F	1.79±0.69	**0.015** *
					M	1.66±0.71	

F: Female; M: Male; Mann Whitney U test applied

*p < 0.05 has been taken as significant.

There were significant associations between year of study and 10 out of 13 coping strategies (Table 7). Year 1 students relied more on “planning” and year 2 on “active coping”, “positive reframing”, and “religion” as preferred coping strategies compared to students from other years. Year 3 students chose “venting” and year 4 students used “use of instrumental support”, “humor”, “self-blame”, “denial”, and “behavioral disengagement” preferred coping strategies more than students of other years. There were statistically significant associations between the year of study and 19 out of 26 COPE items. It is interesting to note that the highest mean item score of COPE strategy items related to “religion”: “I’ve been trying to find comfort in my religion or spiritual beliefs” and “I’ve been praying and meditating” were chosen by year 2 students compared to students of other years (Table 7).

**Table 7:** Comparison of year of study with COPE domains and items.

COPE domain	Year of study	Mean domain score±SD	p value	Coping Strategy	Year of study	Mean item score±SD	p value
**Active coping**	1	2.36±0.51	**<0.001** *	I’ve been concentrating my efforts on doing something about the situation I’m in	1	2.28±0.61	**<0.001** *
	2	2.41±0.38			2	2.35±0.53	
	3	2.11±0.51			3	2.04±0.59	
	4	2.19±0.56			4	2.15±0.68	
				I’ve been taking action to try to make the situation better	1	2.43±0.59	**<0.001** *
					2	2.47±0.50	
					3	2.19±0.58	
					4	2.24±0.59	
**Planning**	1	2.48±0.53	**<0.001** *	I’ve been trying to come up with a strategy about what to do	1	2.43±0.63	**<0.001** *
	2	2.44±0.53			2	2.43±0.58	
	3	2.26±0.52			3	2.19±0.62	
	4	2.23±0.58			4	2.25±0.62	
				I’ve been thinking hard about what steps to take	1	2.53±0.66	**<0.001** *
					2	2.44±0.59	
					3	2.32±0.57	
					4	2.20±0.68	
**Use of instrumental support**	1	1.89±0.64	**0.004** *	I’ve been getting help and advice from other people	1	1.98±0.73	0.094
	2	1.85±0.52			2	1.92±0.58	
	3	1.99±0.62			3	2.01±0.71	
	4	2.07±0.63			4	2.10±0.70	
				I’ve been trying to get advice or help from other people about what to do	1	1.82±0.68	**<0.001** *
					2	1.78±0.59	
					3	1.97±0.61	
					4	2.03±0.63	
**Use of emotional support**	1	1.91±0.66	0.263	I’ve been getting emotional support from others	1	1.89±0.74	0.065
	2	1.87±0.51			2	1.83±0.63	
	3	1.89±0.69			3	1.90±0.74	
	4	2.0±0.69			4	2.03±0.75	
				I’ve been getting comfort and understanding from someone	1	1.94±0.71	0.778
					2	1.91±0.65	
					3	1.89±0.71	
					4	1.97±0.73	
**Positive reframing**	1	2.30±0.65	**0.008** *	I’ve been trying to see it in a different light, to make it seem more positive	1	2.23±0.66	**0.025** *
	2	2.35±0.51			2	2.33±0.63	
	3	2.16±0.64			3	2.11±0.75	
	4	2.16±0.63			4	2.13±0.72	
				I’ve been looking for something good in what is happening	1	2.37±0.73	**0.007** *
					2	2.37±0.57	
					3	2.21±0.68	
					4	2.19±0.69	
**Acceptance**	1	2.31±0.65	0.306	I’ve been accepting the reality of the fact that it has happened	1	2.30±0.77	0.566
	2	2.31±0.56			2	2.29±0.68	
	3	2.26±0.59			3	2.24±0.64	
	4	2.22±0.56			4	2.27±0.63	
				I’ve been learning to live with it	1	2.31±0.71	0.135
					2	2.33±0.56	
					3	2.29±0.66	
					4	2.18±0.65	
**Humor**	1	1.70±0.72	**<0.001** *	I’ve been making jokes about it	1	1.76±0.78	**<0.001** *
	2	1.78±0.68			2	1.91±0.76	
	3	1.83±0.68			3	1.81±0.74	
	4	2.12±0.73			4	2.15±0.77	
				I’ve been making fun of the situation	1	1.64±0.76	**<0.001** *
					2	1.65±0.74	
					3	1.85±0.73	
					4	2.08±0.78	
**Religion**	1	2.55±0.57	**<0.001** *	I’ve been trying to find comfort in my religion or spiritual beliefs	1	2.50±0.69	**0.017** *
	2	2.64±0.44			2	2.63±0.48	
	3	2.45±0.53			3	2.50±0.66	
	4	2.41±0.59			4	2.42±0.62	
				I’ve been praying or meditating	1	2.60±0.60	**<0.001** *
					2	2.64±0.48	
					3	2.39±0.56	
					4	2.40±0.64	
**Venting**	1	1.81±0.53	**0.001** *	I’ve been saying things to let my unpleasant feelings escape	1	1.91±0.69	0.350
	2	1.76±0.59			2	1.84±0.63	
	3	1.96±0.50			3	1.93±0.70	
	4	1.93±0.60			4	1.82±0.74	
				I’ve been expressing my negative feelings	1	1.71±0.67	**<0.001** *
					2	1.68±0.69	
					3	1.99±0.58	
					4	2.05±0.76	
**Self-blame**	1	1.89±0.66	**<0.001** *	I’ve been criticizing myself	1	1.94±0.70	**<0.001** *
	2	1.86±0.72			2	1.76±0.73	
	3	1.96±0.68			3	1.97±0.75	
	4	2.18±0.68			4	2.27±0.73	
				I’ve been blaming myself for things that happened	1	1.83±0.74	**0.011** *
					2	1.96±0.79	
					3	1.94±0.71	
					4	2.10±1.96	
**Self-distraction**	1	2.13±0.63	0.192	I’ve been turning to work or other activities to take my mind off things	1	2.10±0.68	0.033*
	2	2.22±0.59			2	2.22±0.71	
	3	2.29±0.51			3	2.31±0.64	
	4	2.22±0.55			4	2.20±0.66	
				I’ve been doing something to think about it less, such as going to movies, watching TV, reading, daydreaming, sleeping, or shopping	1	2.16±0.76	0.766
					2	2.23±0.65	
					3	2.26±0.62	
					4	2.23±0.62	
**Denial**	1	1.46±0.59	**0.005** *	I’ve been saying to myself ‘this isn’t real’	1	1.48±0.71	**0.008** *
	2	1.64±0.64			2	1.59±0.65	
	3	1.60±0.68			3	1.64±0.75	
	4	1.71±0.69			4	1.70±0.72	
				I’ve been refusing to believe that it has happened	1	1.44±0.65	**0.001** *
					2	1.68±0.69	
					3	1.56±0.70	
					4	1.72±0.76	
**Behavioral disengagement**	1	1.66±0.57	<0.001*	I’ve been giving up trying to deal with it	1	1.64±0.65	**<0.001** *
	2	1.54±0.58			2	1.58±0.64	
	3	1.83±0.65			3	1.81±0.71	
	4	1.95±0.65			4	1.92±0.72	
				I’ve been giving up the attempt to cope	1	1.68±0.68	**<0.001** *
					2	1.50±0.65	
					3	1.85±0.64	
					4	1.98±0.69	

Kruskal Wallis test applied

*p < 0.05 has been taken as significant.

## Discussion

For undergraduate programs to undergo transformation, validated data about dentistry students’ educational experiences is necessary. Advanced dental education and training are growing increasingly popular worldwide, and for many dental students, it represents a pivotal moment in their career. Young professionals face particular problems, such as juggling several clinical and academic obligations. As high and prolonged stress have been linked to burnout, it should be taken very seriously [10]. Pakistani undergraduate dental students report high degrees of academic burnout [25] and high levels of stress [2, 26].

For the majority of the stress-related items, the students reported moderate levels of stress as these responses were in the range of 2 and 2.99 followed by items which were reported to be highly stressful. Similar results were reported by others in another study conducted in Islamabad, Pakistan where majority of the students were ‘moderately stressed’ [8]. Therefore, it could be speculated that these students were enrolled in a moderate to highly stressful educational environment. This also suggested that the educational environment in these dental schools may have adverse effects on the well-being and psychological health of these students. On the contrary, in a multi-country study, the overall stress scores in majority of the domains were ‘mildly stressful’ in most of the items of DES questionnaire [27]. This could be because of differences in the geographical location, curriculum, and educational environment in different countries.

It was interesting to note that the highest mean domain score was of “workload” which corroborates with others [27]. Some authors have reported varied results. In Nepal, undergraduate dental students felt items relating to clinical training were perceived as highly stressful by the students [28]. In another study conducted in Syria the authors reported highest mean scores were of patient treatment/interaction and clinical training domains [29]. This information adds to the knowledge base of most stressful items in undergraduate dental training in Pakistan. The results from these findings indicate that undergraduate students feel more responsible towards their degree requirements. These areas need to be addressed by the curriculum developers and policy makers. In general, addressing the problems faced by undergraduate dental students cannot be over emphasized as these young professionals often serve as ‘role models’ for society.

The lowest mean domain score was that of “faculty and administration”. Others have reported varied results where social stressors domain had the lowest mean score [27]. This could be because of differences in graphical location, and social and financial pressures in our study population. Interestingly, the least stress scoring item “language barrier” of the questionnaire belonged to “self-efficacy domain”. Similar results were reported in another study from Islamabad, Pakistan [8]. As the medium of education in undergraduate dental schools is English, thus the finding that students reported less scores in “language barrier” compared to other stress items is understandable. It may also suggest that students had confidence on their abilities of English language. Dentistry in undergraduate is taught in English language with almost all the reference material available in this language. Thus, students with good command on the language would be less stressful due to language barrier.

In this study, “lack of time for relaxation” and “overloaded feeling due to huge syllabus” were perceived as highly stressful by majority of the students in “workload” domain of DES questionnaire. Similar results were reported by others in a dental school in Islamabad, Pakistan where the authors reported that “syllabus load” and “lack of relaxation time” were perceived as stressful or highly stressful by majority of their dental students in one dental school [8]. In a study conducted in UK, “lack of time for relaxation” was among the top 10 stressors labelled as highly stressful by 30.5% of the dental undergraduate students and “examinations and grades” was considered highly stressful by 72.3% of the students [30]. The difference in the taught curriculum, educational environment, and financial responsibilities could be the reasons of these results. In the domain of “self-efficacy beliefs” item “fear of failing a course or the year” scored higher than other items in our study participants. This corroborates with previous studies [8,30]. In “patient treatment/interaction” domain item “fear of dealing with patients who do not disclose the existence of a contagious /co-existing disease” scored higher than other items. In another study, similar results were reported [8].

Female students had higher mean domain DES scores and perceived more stress compared to male dental students in all the stress domains. Similar results were reported by others that females perceive stress more compared to male dental students in majority of the domains [8,17,27,31–33]. In postgraduate dental students in India, significant associations were reported between sex and “self-efficacy beliefs” and “personal and accommodation factors” [34]. In a study conducted in Peru, authors reported that sex was not associated with mean domain or item scores of DES questionnaire [35]. The difference could be because of different demographics and social norms. This could also be because female students are more concerned about making their mark, proving themselves, and taking responsibilities seriously which is making them more stressed compared to male students in our society [36].

Female students were more stressed because of “performance pressure”, “patient treatment/interaction”, and “clinical training” than male dental students. As apparent from the increased females to male ratio in dental schools in Pakistan because admission criteria is based on merit, female students could be more focused on their studies and on developing skills required in future to earn in general dental practice field and to compete for postgraduate spots. Female students may feel that for having their impact known and appreciated by the society at large, they have to make extra efforts to compete with males in the field which is also apparent from the results that females had significantly higher stress scores for items “fear of not being able to join a post-graduate dental education program” and “insecurity concerning lack of employment positions” [37,38]. With current increase in inflation, females may also feel more responsible towards sharing the financial burden of their families causing an increased stress perception. In Pakistan, there are limited employment opportunities available after undergraduate studies in public and private set ups compared to the number of graduates each year which is an area that needs to be evaluated by Government bodies to create positions for these graduates to reduce their stress about future job prospects.

In this study, there were significant associations between the year of study and most of the DES domains. Similar results were reported by others [8,17,27,33]. Domain-wise DES analysis revealed that year 4 dental students had higher mean stress scores in the majority of the domains compared to students of other years especially related to “workload”, “self-efficacy beliefs”, “faculty and administration”, “clinical training”, and “social stresses”. Similar results were reported by others that senior clinical year students have higher mean stress scores compared to pre-clinical year students [27]. In a study in Chinese students, higher and statistically significant mean scores were reported in year 3 and year 4 students compared to year 1 and year 2 students in “workload” and “self-efficacy” domains only [39]. However, the authors did not include “patient treatment/interaction” and “clinical training” domains of DES questionnaire in data collection.

It was interesting to note that in this study, year 1 students were more stressed due to “dealing with patients with dirty mouths” whereas in pre-clinical years in Pakistan, students are not involved in patient dealing. It could be because these students are more apprehensive about their future clinical training and patient dealing while in their pre-clinical years. Similar results were reported in a study on Romanian dental students in which authors reported that freshman students were more stressed about clinical responsibilities compared to senior dental students [40]. This might be the result of a discrepancy between what students expect from dentistry school and what they actually experience, and the emotional strain brought on by the discrepancy must have increased the students’ psychological stress [41]. Furthermore, first- and final-year students generally have higher levels of stress than middle-year students [42] which is also evident from year 4 students being more stressed in most of the stress items in this study.

In the domain of “workload”, year 1 students have higher mean item scores compared to other years of the items “amount of assigned class work”, “lack of time to do assigned work”, and “overloaded feeling due to huge syllabus”. Similar results were reported by others [43,44]. This can be explained by the fact that students have a lot of academic workload of the subjects which were completely different form their secondary school subjects with introduction of new terminologies and concepts, increase number of written and oral assessments relating to subjects of Anatomy, Physiology, Biochemistry, Oral Biology, and Tooth Morphology. These students are still adapting to their new environment of dental school besides working on their academics.

Year 4 students were more stressed than other students in the majority of the stress items. In clinical years as students are approaching graduation, they need to learn the skills to pass their clinical assessments and also required to complete their assigned clinical cases in each clinical subject during rotations in these departments resulting in late hours and less time in between the clinical sessions for relaxation. Similar results were reported by others [39]. It is also evident from increased stress scores of “lack of confidence to be a successful dental student”, “fear of not being able to join a post-graduate dental education program”, and “insecurity concerning professional future” items in these year 4 students compared to other years that these students are not very hopeful about their future prospects in finding suitable employment or a place in highly competitive postgraduate programs which is also evident from increased stress scores in “financial responsibilities” of social stresses domain as well. In a study conducted in Peru, authors reported that clinical year students were more stressed compared to pre-clinical year dental students [35]. However, they reported that year 1 students were more stressed compared to year 2 students in the pre-clinical years, whereas, year 2 students were more stressed compared to final year dental students in clinical years. In another study from Islamabad, Pakistan, authors reported that year 3 students were more stressed compared to students from other years in “shortage of allocated laboratory time”, “shortage of allocated clinical time”, “difficulty in learning precision manual skills required in pre-clinical work”, “responsibility of getting suitable patients”, “working on patients with dirty mouths”, and “clinical requirement (quota)”. This difference could be because of different sample sizes and study population demographics.

The year 4 students who are about to graduate but feel stressful because of “lack of confidence in own decision making” and “lack of confidence to be a successful dentist” are rather concerning and shows that the graduating students are not yet confident in their clinical skills. Similar results were reported in a study conducted on Chinese dental students where a gradual increase in DES scores in performance pressure domain were reported from year 1 to year 4 dental students [39]. Lack of confidence could be because these students feel they are not properly trained or did not have enough clinical training or exposure to be confident enough to run their own dental practices.

Year 4 students were also more stressed in all of the items related to “faculty and administration” domain. Higher stress scores were also reported in Chinese dental students for “lack of approachability of staff”, “lack of input into the decision-making process of the school”, “rules and regulation of dental school”, and “amount of cheating in dental school” [39]. This suggests that with the increase in student-patient interaction and clinical training, the expectations of faculty members and patients also increase, and students are required to meet a set standard for the graduating class for the required clinical skills and academics. It also highlights that the faculty to student ratio must be increased in clinical years so that teaching and learning of clinical skills is not compromised as students are stressed because of this element.

When comparing the COPE domains, in this study, the question about finding solace through religious or spiritual beliefs had the highest mean score “I’ve been trying to find comfort in my religion or spiritual beliefs” and “I’ve been praying and meditating”, suggesting that undergraduate dental students were using these coping approaches the most frequently. It is consistent with earlier research in which undergraduate dental and medical students reported using “religion” as a frequently used coping mechanism [2,6,45–47]. On the contrary, in the study in British Columbia, “religion” was the least commonly used coping domain among the adaptive coping strategies [48]. This could be because most of the study participants in the study were non-religious and from different ethnic backgrounds compared to our study.

It was also encouraging to note that items such as “I’ve been saying to myself this isn’t real” and “I’ve been refusing to believe that it has happened” related to “denial” scored the lowest, implying that the undergraduate dental students had greater self-assurance and were more realistic. This is in accordance with previously published study on medical students [47]. The questions in the COPE-strategy that dealt with coping tactics “behavioral disengagement” and “denial” had the lowest scores overall. This suggested that our undergraduate dental students were not as likely to be utilizing avoidance-focused coping mechanisms, which are typically linked to students who are under a lot of stress [30,49]. This is also consistent with earlier research on undergraduate dental and medical students’ selection of positive or adaptive coping strategies [6,46]. In a study conducted in British Columbia in which majority of the students were Asian, undergraduate dental students frequently used coping strategies were different. Authors reported “self-distraction”, “positive reframing”, “use of emotional support”, and “active coping” were the most frequently used coping strategies in that order based on mean scores [48]. In their study, maladaptive coping strategy “self-distraction” were the most commonly used strategy compared to other adaptive coping strategies.

After “religion”, the second COPE domain with higher mean domain score was “planning”. In a prior study, “planning” was also discovered to be the most often employed coping technique among Turkish undergraduate dental students [22,50] and in medical students [51]. Additionally, a study conducted on Saudi undergraduate dental students also revealed that the students’ typical coping mechanisms included “planning” and “religion” [6]. Similar results were reported in dental students from Malaysia [46]. This implies that in our undergraduate dental students, spirituality and planning are considered as preferred coping mechanisms. Religious participation is linked to mental and psychological welfare, according to studies, because many faith teachings promote positive behavior and prohibit negative conduct and mitigates daily stress and improves general well-being [52]. In a recent study conducted on students from Lahore, Pakistan from non-medical/dental educational programs have also stated that adaptive coping strategies “religion”, “acceptance”, “self-distraction”, and “active coping” were the commonly adopted coping strategies [45]. It can be implied that dental students also use more constructive coping strategies in Pakistan, similar to other students which could be due to similar social and cultural practices.

There were differences in the ways that undergraduate female and male dental students were managing their stress. This is comparable to earlier research on undergraduate dental and medical students, which similarly discovered differences in the kinds of coping techniques the students employed [6,22,46,50,53]. Moreover, significant associations between sex and adaptive coping strategies were noted including “active coping”, “religion”, and “positive reframing”, and females were using these coping strategies more compared to male dental students. Higher COPE scores for “religion” were also reported by others in females compared to males [53]. On the contrary some authors have reported that there is no association between these adaptive coping strategies and sex [31]. However, they did report higher COPE mean scores in females compared to males of COPE items “active coping” and “religion”.

Detailed analyses of the items of these COPE domains revealed interesting results. Females were more engaged in active coping strategies by making efforts to improve their current situation and to make their situation better compared to males. Females were more engaged in strategically planning to do something to improve their situation and were more optimistic by focusing on something positive from the situation compared to male dental students. Similar results were reported by other [53]. Females also had more acceptance by trying to adapt to the situation compared to males. Similar results were reported by others in postgraduate dental students in India [34]. Females were also more inclined towards religion and were finding solace in prayer and meditation compared to males.

It is noteworthy to report that these dental students were not relying on emotional and instrumental support as the associations were not significant between sex and these coping strategies. Similar results were reported by others [6,31]. On the contrary, significant associations were reported between sex and these items in Turkish dental students [22,50], Polish dental students [53], and in US college students [54]. This could be because of different study populations and educational environments. This may also imply that for this generation of Pakistani students, emotional support from others or getting advice and help from family or friends may not be suitable methods for them to cope with stressful situation.

Furthermore, in this study, there were also significant associations between sex and the following maladaptive domains “denial”, “venting”, “behavioral disengagement”, and “self-blame”. In these domains, mean COPE scores were more for females compared to male dental students implicating that the females were relying more on maladaptive coping strategies compared to males. Similarly, some authors have also reported higher mean scores in female students compared to males in “venting” and “behavioral disengagement” but higher scores in male students in “denial” and “self-blame” domains reporting only “denial” as significantly associated coping strategies [31]. Others have also reported that females turned more often to “venting of emotions” and “blaming themselves” [53]. Saudi female dental students also relied more on using “denial” and “self-blame” as preferred coping strategies compared to males [6].

A detail analyses of these individual coping strategies reveal that females were relying more on distraction by thinking less of the situation using support such as media, reading, daydreaming, sleeping, or shopping compared to male dental students. Similar results were reported by others in medical students [14,46]. Females were also considering the situation as unreal or refusing to believe it to cope with their situation compared to males. Females were also venting more by allowing unpleasant feelings to escape or by expressing their negative emptions compared to males. The results corroborates with earlier research [53]. Females also reported that it was easier to give up in their attempt to cope compared to males. Females were also self-criticizing and self-blaming themselves more compared to males in order to cope with stressful situation. These results are in accordance with previously published studies in Polish dental students [53].

In this study, there were significant associations between year of study and 10 out of 13 coping strategies. Similar results were reported by others for “planning” “denial”, “venting”, and “positive reframing” [22,50]. On the other hand, in dental students from India, significant associations were reported for “active coping”, “planning”, “emotional support”, “instrumental support”, and “behavioral disengagement” coping strategies [31]. This suggests that coping strategies choices vary to some extent with educational environment, social factors, and demographic location.

In this study year 1 students relied more on “planning” as a coping strategy compared to students from other years. On the contrary, Turkish pre-clinical dental students from year 2 preferred this strategy compared to year 1 students who used “venting” more often [50]. In the study in India, year 1 students preferred “religion”, “emotional support”, and “instrumental support” compared to students of senior years [31].

In this study, it was interesting to note that “religion” and “positive reframing” were the most commonly used coping strategies by year 2 students compared to students from other years. Similar results were reported by others [50]. “Active coping”, “planning”, “humor”, and “self-blame” were the coping strategies more commonly used by year 3 students from India compared to students of other years, however, associations with “humor” and “self-blame” were not statistically significant [31].

Year 3 students chose maladaptive coping “venting” and “self-distraction” as preferred coping strategies compared to students from other years which is in accordance with previously published studies [22]. Others have reported that adaptive coping strategy “positive reframing” was more commonly used by year 3 dental students, though the association between year of study and this domain was not statistically significant but mean scores were higher in year 3 students compared to students of other years [31].

In this study, year 4 students commonly used adaptive coping strategies namely “humor” and “use of instrumental support”, and maladaptive coping strategies “denial”, “behavioral disengagement”, and “self-blame” more often than students of other years of study. Some authors have also reported that senior clinical year dental students of Turkish dental school also used “humor” and “denial” coping strategies compared to junior clinical dental year students [22]. In the study on Indian dental students, “denial” and “venting” were most commonly used by year 4 students, though the associations were not significant for these maladaptive coping strategies [31]. In this study instrumental support and emotional support were not significantly associated with year of study. Similar results were reported by others [22,50].

This study provides valuable insights into the lives of undergraduate dental students. However, the results of this study should be used with caution. Possible inaccurate responses could be caused by misinterpretation of survey questions, students rushing through the survey, and students who would not take the survey seriously. Moreover, there were more female participants than male participants because the majority students were female in these dental schools which could have caused selection bias. Despite the limits of this study, the findings have helped to clarify the elements that can lead to stress and the coping strategies that dental students use to get through difficult circumstances during their undergraduate studies. This information makes the study particularly useful for understanding the educational experiences of Pakistani undergraduate dental students, the kinds of stressors they face, and the coping mechanisms they use. It is especially helpful for policymakers, curriculum developers, medical educationists, psychologists, psychiatrists, teachers, student counselors, and other support networks.

## Conclusion

It is concluded that sex and year of study can influence the extent of perceived stress and choice of coping strategies employed to overcome stressful situations. Problem-focused, emotion-focused, and avoidant-focused coping strategies are used to overcome perceived stressors of the educational environment. For the emotional, psychological, and professional well-being of undergraduate dental students, dental schools must provide a nurturing and positive learning environment implementing strategies for stress prevention and management.
